# Changes in Adipokine, Resitin, and BDNF Concentrations in Treatment-Resistant Depression after Electroconvulsive Therapy

**DOI:** 10.3390/brainsci13101358

**Published:** 2023-09-22

**Authors:** Agnieszka Permoda-Pachuta, Magda Malewska-Kasprzak, Maria Skibińska, Krzysztof Rzepski, Monika Dmitrzak-Węglarz

**Affiliations:** 1 Department of Psychiatry, Medical University of Lublin, 20-439 Lublin, Poland; 2 Department of Psychiatry, Poznan University of Medical Sciences, 60-572 Poznan, Poland; 3 Department of Psychiatric Genetics, Poznan University of Medical Sciences, 60-806 Poznan, Poland; 4 Mental Health Center at the HCP Medical Center, 61-485 Poznan, Poland

**Keywords:** depression, electroconvulsive therapy, adipokines, candidate biomarkers

## Abstract

Objectives: One of the current challenges in psychiatry is the search for answers on how to effectively manage drug-resistant depression. The occurrence of drug resistance in patients is an indication for the use of electroconvulsive therapy (ECT). This method is highly effective and usually results in relatively quick health improvement. Despite the knowledge of how ECT works, not all of the biological pathways activated during its use have been identified. Hence, based on the neuroinflammatory hypothesis of depression, we investigated the concentration of two opposite-acting adipokines (anti-inflammatory adiponectin and proinflammatory resistin) and BDNF in antidepressant-resistant patients undergoing ECT. Methods: The study group comprised 52 patients hospitalized due to episodes of depression in the course of unipolar and bipolar affective disorder. The serum concentration of adipokines and BDNF was determined before and after the therapeutic intervention using an ELISA method. In the analyses, we also included comparisons considering the type of depression, sex, and achieving remission. Results: Adiponectin, resistin, and BDNF concentrations change after ECT treatment. These changes are correlated with an improvement in the severity of depressive symptoms and are more or less pronounced depending on the type of depression. Conclusions: Although not all observed changes reach statistical significance, adipokines in particular remain exciting candidates for biomarkers in assessing the course of the disease and response to ECT treatment.

## 1. Introduction

The etiology of depressive disorders, despite intensive research, is still not fully understood. Updated World Health Organization (WHO) reports indicate that about 3.8% of the world’s population suffers from depression. It has been estimated that depression is the most common factor leading to disability and is among the leading causes of suicide attempts and deaths. Therefore, depression and suicide are among the priority conditions covered by the WHO [1]. Primary biological hypotheses indicate a disruption of noradrenergic, dopaminergic, and serotonergic neurotransmission [2].

In addition, the inflammatory hypothesis is central to the development and course of depression [3]. The first findings that depression is characterized by cellular immune activation and inflammation were presented as early as the 1990s [4]. Further studies have confirmed altered numbers and activity of immune cells from lymphocytes and monocytes to macrophages [5,6]. Depressed patients experience increased proinflammatory cytokines, particularly interleukin 1β (IL-1β), IL-2, IL-4, and IL-6 [7]. Cytokines cross the blood–brain barrier (BBB) by acting on different areas of the CNS. It is believed that cytokines can modify the metabolism of monoamines (i.e., dopamine, norepinephrine, and serotonin) in the midbrain nuclei and, through direct and indirect pathways, can induce excessive cortisol secretion—by stimulating the stress axis and altering the sensitivity of glucocorticoid receptors [8]. Thus, higher concentrations of inflammatory cytokines stimulate the HPA axis and trigger a physiological stress response [9]. Abnormal functioning of the HPA axis is seen in 50–75% of patients with depression [10]. Enhanced functioning of the HPA axis and impaired immune system cause abnormal functioning of the so-called kynurenine pathway. This pathway transforms tryptophan into two compounds responsible for mood regulation: serotonin and melatonin [11]. Inflammatory factors lead to increased activation of the enzyme indoleamine-2,3-dioxygenase (IDO) in neurons, astrocytes, and microglial cells. The IDO enzyme converts tryptophan into neurotoxic kynurenine, which increases the likelihood of neurodegenerative and neurotoxic processes. Consequently, the level of tryptophan required for serotonin production decreases, which plays a significant role in the etiology of depressive states [12]. For a detailed discussion of the neuroinflammatory hypothesis and the kinesin pathway, the reader can refer to a comprehensive study [13].

The hippocampus is exceptionally sensitive to chronic stress and impaired regulation of the HPA axis, where there is decreased expression of brain-derived neurotrophic factor (BDNF), reduced efficiency of synaptic conduction (impaired long-term potentiation), and arrested neurogenesis [14]. Herein lies the inclusion of another neurotrophic theory relevant to depression related to differentiation, neuronal growth, neurogenesis, modulation of plasticity, and neuroregeneration. BDNF, nerve growth factor (NGF), and neurotrophin 3 are involved in the processes of neuroplasticity and neurogenesis, exerting essential effects on the functioning of both the adult and developing brain, and their expression is significantly regulated by stress and psychotropic drugs. A recent study found an association between low BDNF levels, high levels of inflammatory markers, and the development of depressive symptoms [15]. An increased serum BDNF level was observed after treatment with antidepressants and after ECT procedures. Based on this, it was concluded that it might be a promising biomarker of depression [16].

The cited data combine to form a common neuroinflammatory hypothesis [17]. In addition to classical cytokines secreted by immune cells, proteins other than classical cytokines associated with inflammation also fit into this theory. Of particular interest seem to be adipokines secreted by white adipose tissue—an important and still underestimated endocrine organ [18]. Adipokines regulate appetite and satiety, body energy homeostasis, and carbohydrate and fat metabolism. They participate in coagulation, angiogenesis, and vascular remodeling, including atherosclerotic plaque formation, and regulate blood pressure. In addition, they exert effects on immune and reproductive system function and osteogenesis. Adipokines are active at the central, tissue, and cellular levels, exhibiting endo-, para-, and autocrine effects [19]. Letho et al., 2010 pointed out the association with depression of two adipokines with opposing effects—the anti-inflammatory adiponectin and the proinflammatory resistin [20].

Adiponectin exerts its effects through the activation of specific AdipoR1 and -R2 receptors. These receptors are characterized by high levels of expression in various brain regions, including those associated with mood regulation (e.g., the hippocampus). Adiponectin exerts its anti-inflammatory effects by increasing the expression of anti-inflammatory cytokines such as interleukin 10 (IL-10) while decreasing the expression of tumor necrosis factor-α (TNF-α) [21]. Despite the crucial functional role of adiponectin in the CNS, studies on adiponectin levels in patients with depression are few and inconclusive. Some reports indicate its concentrations are higher [22], lower [23], or unchanged [24] in depressed patients compared to healthy subjects.

The second intervening adipokine is resistin, which stimulates the secretion of proinflammatory cytokines by macrophages and monocytes. Resistin promotes the secretion of TNF-α and interleukins (IL)-1β, -6, -8, and -12, the production of reactive oxygen species (ROS), and the inhibition of endothelial nitric oxide synthase (eNOS). Resistin also stimulates the release of monocyte chemotactic protein-1 (MCP-1) and nuclear factor kappa-enhancer light chain activation of activated B cells (NF-κB) [25]. Meta-analyses have shown that resistin levels are lower in depressed patients [26] and remain lower even after antidepressant treatment than in healthy controls [27]. In contrast, recent studies have suggested that higher serum resistin levels are associated with MDD pathophysiology and may be an early indicator for assessing MDD risk [28].

In the context of the few inconclusive results available, studies of adipokines in depression must continue.

Despite the advances that have been made in pharmacotherapy, there are still about 20–40% of patients with a major depressive episode (both unipolar and bipolar) who do not show a clinical response to current treatment with antidepressants [29,30].

Electroconvulsive therapy (ECT) is a method that has been known and used for more than 80 years with proven therapeutic efficacy in some mental illnesses. ECT remains the most effective option for patients with treatment-resistant depression (TRD) [31]. Although the basic biological mechanism of action of ECT related to the induction of a seizure is known, not all the biological pathways activated during the application of the treatments have been identified [32]. It is known that during the treatments, among other things, there is a rapid release of transmitters in the CNS, an enhancement of the polarity of neurolemma, an increase in the activity of receptors and their affinity for transmitters (which, in perspective, facilitates the selection of drug treatment in a drug-resistant patient after a series of ECT treatments), and the previously disturbed hypothalamic–pituitary–adrenal axis is regulated. There is an increase in the permeability of the blood–brain barrier [33]. Performing ECT treatments is costly and carries certain risks for the patient [34]. Although patients are appropriately qualified and somatically tested before undertaking such treatment, the testing is routine and more aimed at safety than a thorough understanding of a given patient’s functioning biology. Thus, for many years, using the resources of biological knowledge and the pathophysiology of mental illness in patients, biomarkers of the efficacy of this therapy have continued to be sought to more precisely determine its suitability for treating a given patient, ensure safety, and reduce costs. 

## 2. Aim of the Study

In the present study, we performed replication measurements of peripheral concentrations of adiponectin, resistin, and BDNF in a homogeneous group of patients hospitalized for a depressive episode. We completed the comparative analysis focused on the state before (pre-T) and after electroconvulsive therapy (post-T). Associations of selected biomarkers with the type of depression, sex, and severity of depressive symptoms were also analyzed.

We hypothesized that the concentrations of carefully selected proteins change after ECT, that these changes can be captured and monitored using standard assay methods, and that they can predict the achievement of improvement in disease symptoms.

## 3. Methodology

### 3.1. Participants

The study included patients from the Polish population hospitalized in the Department of Adult Psychiatry at the Poznan University of Medical Science (PUMS). All patients met the criteria for treatment-resistant depression (TRD) in the course of recurrent depressive disorder (ICD-10: F33, N = 29) or bipolar affective disorder (ICD-10: F31, N = 25). For simplicity, we will use the terms unipolar (UD) and bipolar depression (BD) hereafter. TRD was defined as a lack of improvement after at least two adequate courses of antidepressant and/or mood-stabilizing treatment (including augmentation) used in proper doses within the previous three months [35]. In all patients, ECT treatment was chosen as the treatment modality.

### 3.2. Inclusion and Exclusion Criteria

In the study, the inclusion and exclusion criteria developed by the Biological Section of the Polish Psychiatric Society for ECT treatments were applied [34,35,36]. The key contraindication is the coexistence of certain serious general diseases. These include epilepsy, other severe brain diseases (e.g., encephalitis, diseases with increased intracranial pressure, recent stroke), severe heart diseases (up to six months after myocardial infarction), significant hypertension, severe clotting disorders or significant anemia, advanced osteoporosis, aortic aneurysms, thrombophlebitis, or some ophthalmological diseases. For women, the exclusion criteria are pregnancy and breastfeeding. In addition, a BMI of ≥25.0 kg/m^2^ was used as an additional exclusion criterion because of the effect of excessive body weight on adipokine synthesis and release [37]. The list of inclusion and exclusion criteria is shown in Table 1.

The Hamilton Depression Rating Scale (HDRS17) was used to assess the severity of depression symptoms. A score > 18 was required for inclusion in the study, indicating at least moderate severity of depression. A score ≤ 7 was required for obtaining clinical remission or at least a 50% reduction at HDRS, defined as treatment response. On this basis, we distinguished patients with recovered (rec) and non-recovered (non-rec) status.

The study group comprised 54 inpatients (15 male, 39 female) aged 21–82 (mean 54 ± 12.8) years. The mean duration of the illness was 15 ± 18.5 years, and the current depressive episode was 35 ± 65 weeks (Table 2).

During ECT treatment, the patient’s treatment protocol was not interfered with. Patients took medications as indicated and under the control of the treating physician. Among the patients studied, ten patients took atypical neuroleptics, five patients took antidepressants, and thirty-eight patients took both groups of drugs. Due to the variation in age and pharmacotherapy used, we analyzed the effect of these variables on the concentrations of the biomarkers studied, showing no significant relationship (*p* < 0.05) (Tables S1 and S2).

### 3.3. ECT Procedure

Each patient, before ECT, underwent anesthesiological and cardiological consultation to qualify for the treatment procedure. Before ECT, anticonvulsants and lithium carbonate were discontinued in all patients. Most patients were maintained on their current antidepressants with a reduced dose. Patients received stable doses of medications for approximately two weeks before the first ECT intervention. Thiopental at a dose of 2–5 mg/kg body weight or ketamine 1.0–1.5 mg/kg body weight were alternatively used for general anesthesia. The subjects received between 6 and 16 treatments, the number of which was individually selected by the treating physician. The Thymatron System IV device was used for the treatments. Bilateral placement of electrodes in the frontal and temporal regions was used. The administered current energies ranged from 101.8 to 506.0 mC at a constant current value of 900 mA. After each treatment, the clinical condition and response to the treatment were evaluated. The criterion for a complete response to the administered electrical charge was a seizure lasting at least 20 s. EEG electrodes were used to assess bioelectrical function, which was applied bilaterally to the frontal and mastoid regions [39].

### 3.4. Serum Biomarker Determination

Venous blood was collected on morning admission (07:00–08:00 h) from overnight fasting at two time points: pre-treatment (pre-T)—one day before the therapeutic procedure—and post-treatment (post-T)—a day after the completed therapeutic procedure. The serum was immediately separated from the blood by centrifugation at 1000× *g* for 15 min at 4 °C, aliquoted into Eppendorf tubes, frozen at −70 °C, and subsequently assayed.

For the quantitative determination of the studied proteins in blood serum, we used the enzyme-linked immunosorbent assay method (ELISA) using commercially available assays:

- Adiponectin human ELISA kit, cat. no. DEE009 (Demeditec Diagnostics GmbH, Kiel, Germany). The standard curve ranged from 2 to 100 ng/mL. Detailed assay procedure available at: https://www.demeditec.com/en/products/adiponectin-human-elisa-dee009/ifu-dee009-adiponectin-elisa-ce-15a-02-21-m.pdf (accessed on 20 September 2023),
- Human Resistin Quantikine ELISA kit, cat. no. DRSN00 (R&D Systems, Minneapolis, MN, USA). The standard curve ranged from 0.156 to 100 ng/mL. Detailed assay procedure available at: https://resources.rndsystems.com/pdfs/datasheets/drsn00.pdf?v=20230907&_ga=2.19695423.602324339.1694078552-1355354367.1694078552 (accessed on 20 September 2023),
- DuoSet human BDNF, cat. no. DY248 (R&D Systems, Minneapolis, MN, USA). The standard curve ranged from 15.6 to 1000 pg/mL. Detailed assay procedure available at: https://resources.rndsystems.com/pdfs/datasheets/dy248.pdf?_ga=2.191597229.602324339.1694078552-1355354367.1694078552 (accessed on 20 September 2023).

The protocols were performed according to the manufacturer’s recommendations. During verification and optimization of assays, optimal serum dilution for BDNF of 1:150 was determined. Optical density was read using a spectrophotometric reader (Biochrom Asys UVM 340, Cambridge, UK) for a wavelength of 450 ± 10 nm immediately after stopping the color reaction. All determinations were performed in duplicate. The average of both measurements was used for statistical evaluation. Test sample concentration was calculated using a 4-parameter algorithm (4-parameter logistic). Permitted intra- and inter-assay variability was <5% and <10% CV for each studied protein.

### 3.5. Statistical Analysis

Measurable variables were described by arithmetic mean and standard deviation. The normality of the distribution was tested using the Shapiro–Wilk test and the equality of variances was checked using Levene’s test. Due to the lack of normality in the distribution of variables, non-parametric tests were used for statistical analyses:

- Wilcoxon’s paired order test—to test the significance of the difference in the level of the studied parameters at the time before and after electroconvulsive therapy (dependent samples),
- Mann–Whitney U test—to test the significance of the difference in the level of the studied parameters in two groups (independent samples),
- Spearman’s rank correlation coefficient test—to test the correlation between variables.

Multiple linear regression analysis was used to predict improvement in depressive symptoms based on adiponectin, resistin, and BDNF levels.

A minimum sample size was estimated for the population of patients receiving ECT therapy (<1%) [40]. For the assumed confidence level of 0.95 (α = 0.05) and the size of the acceptable estimation error (e ≤ 0.15), the minimum sample size was N = 48.

In the calculation of the size effect, 95% confidence intervals and Glass rank biserial correlation coefficient (r_g_) (for Mann–Whitney U test) and matched-pairs rank biserial correlation coefficient (r_c_) (for Wilcoxon’s test) were used. The values of the coefficients mean a weak (0–0.3), medium (0.31–0.5), strong (0.51–0.7), or very strong effect (0.071–1.0).

The value of *p* < 0.05 was considered statistically significant. Statistical calculations were performed using the STATISTICA 10 PL statistical package.

### 3.6. Ethical Approval

The study followed the rules of the Declaration of Helsinki and complied with Good Clinical Practice guidelines [41]. The Bioethics Committee at the Poznan University of Medical Sciences (PUMS) (resolution No. 434/15, No. 1118/16, No. 295/21) approved the study protocol. All patients were informed about the aims and methods of the research and gave their written consent. Patients participated in the study voluntarily, with the possibility of withdrawing from it at any stage of its duration and without affecting further treatment.

## 4. Results

Demographic and clinical characteristics, adipokines, and BDNF concentrations in depression patients before and after electroconvulsive therapy (ECT) are shown in Table 2. All the studied patients presented significant improvement in the severity of depression symptoms after ECT (*p* < 0.000). Defined recovery status (rec) was achieved by more than 70% of patients treated with ECT. Measurements of the concentrations of individual biomarkers highlighted nominal changes in adiponectin and BDNF (increase) and resistin (decrease) after ECT. However, the observed differences did not exceed the statistical significance threshold (*p* < 0.05).

Since the study included patients with TRD in both UD and BD, we performed a comparative analysis for them (Table 3). Among the biomarkers analyzed, we observed significantly lower resistin levels in patients with UD (*p* = 0.023). Baseline resistin levels were also associated with significantly lower post-treatment change (*p* = 0.015).

In the next step, we compared the biomarkers’ concentrations and their changes between patients distinguished by sex (female vs. male) (Table 4). The only significant difference observed was in the status after ECT treatment (post-T). Women had higher adiponectin concentrations than men (*p* = 0.011). This result may indicate a sex-dependent evolution of this biomarker after treatment.

In the next step, we compared the concentrations of the biomarkers studied and their changes between patients who achieved defined improvement in clinical symptoms (rec vs. non-rec) (Table 5). In the case of adiponectin, we observed significantly higher levels before (pre-T *p* = 0.028) and after treatment (post-T *p* = 0.035) in patients who achieved remission compared to patients who did not. In addition, there was a greater percentage increase in adiponectin levels after therapy in patients without improvement (non-rec *p* = 0.045). Patients without improvement also showed a greater percentage decrease in resistin levels after treatment (non-rec *p* = 0.028).

Table 6 summarizes the results of the Wilcoxon signed-rank test for comparisons of the concentrations of the studied biomarkers in subgroups of patients according to type of depression, sex, and achievement of remission (pre-T vs. post-T). The analyses performed revealed no significant differences.

**Figure 1:**
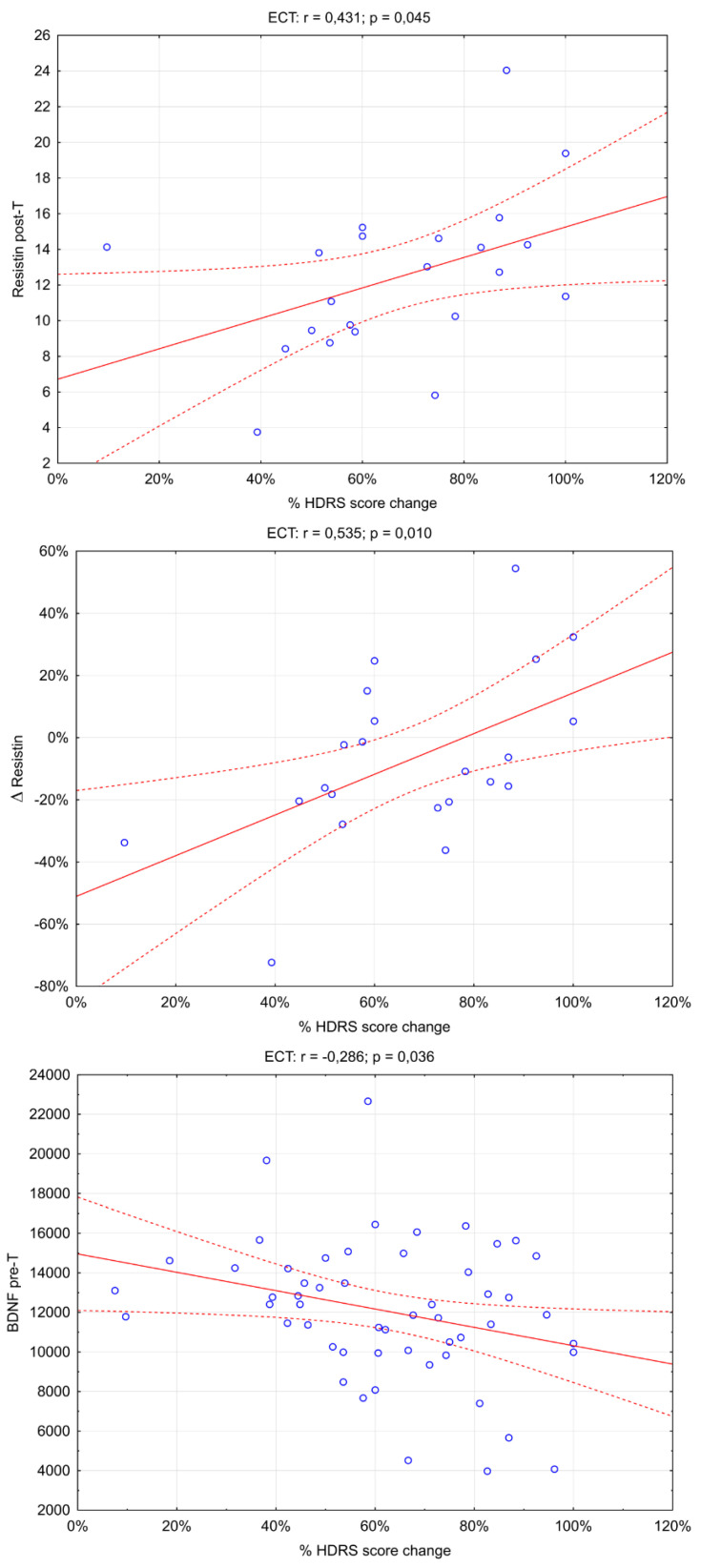
Significant association of adipokines and BDNF serum level with depression symptom improvement. (r_s_: Spearman’s rank correlation coefficient).

An analysis of the association of the concentrations of the biomarkers studied and their changes with the improvement in depressive symptoms, measured as % HDRS score change, was performed (Table S3). There was a significant association of improvement in depressive symptoms with resistin post-T *p* = 0.045, Δresistin *p* = 0.010, and BDNF pre-T *p* = 0.036. Positive correlations exist between % HDRS score change and resistin post-T and Δresistin—Rs = 0.535 and Rs = 0.431, respectively. There is a negative correlation (Rs = −0.286) between % HDRS score change and BDNF pre-T (Figure 1).

**Figure 2:**
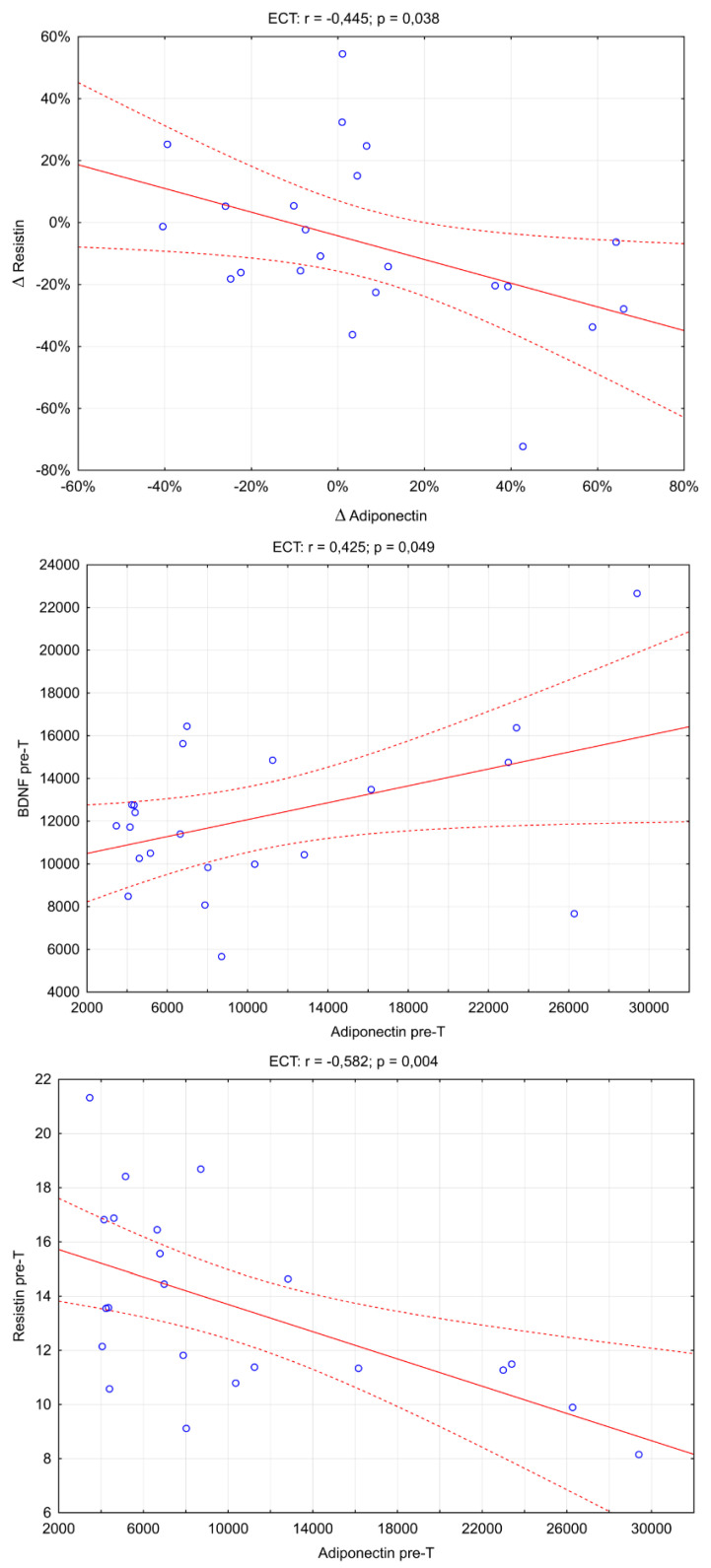
The scatter plot between the adiponectin and the resistin concentrations in the ECT group. (r_s_: Spearman’s rank correlation coefficient).

An analysis of correlations between concentrations and changes in the biomarkers studied was also conducted (Figure 2). There were three significant correlations: adiponectin pre-T and BDNF pre-T (Rs = 0.425; *p* = 0.049), adiponectin pre-T and resistin pre-T (Rs = −0.582; *p* = 0.004), and Δresistin and Δadiponectin (Rs = −0.445; *p* = 0.038).

## 5. Discussion

The study investigated the levels of adipokines and BDNF that are important in the neuroinflammatory theory of depression. Adipokines and BDNF interact with the stress axis and are also synthesized in brain tissue [42]. This study focused on treatment-resistant depression (TRD) treated with ECT, assessing changes in adipokines and BDNF before and after treatment. In the analyses, we also included comparisons considering the type of depression, sex, and achievement of remission of depressive symptoms. The mechanism of the therapeutic effect of ECT is unclear and is still of interest to many researchers [43,44]. The therapeutic effect is related to several mechanisms, including increased release of neurotransmitters in the CNS, enhancement of neuronal polarization, increased permeability of the blood–brain barrier, and stimulation of the HPA axis [45]. The last two mechanisms mentioned above can be linked to the influence of the biomarkers analyzed in this study. Baseline comparisons of adiponectin, resistin, and BDNF concentrations were not significantly different before and after ECT treatment in the entire patient group and subgroups distinguished by depression type, sex, or symptom improvement status. However, an in-depth analysis of the distinguished patient subgroups revealed several correlations, described below.

### 5.1. Adiponectin

For adiponectin, we observed significant correlations such as:

- higher concentrations in women (significant in post-T),
- higher concentrations in patients who achieved an improvement in depressive symptoms (significant in pre- and post-T),
- positive adiponectin concentration correlation with BDNF and negative correlation with resistin of moderate strength (significant in pre-T).

The overall picture from the above correlations indicates that using ECT affects changes in adiponectin concentration (increase). Nevertheless, the strength of these changes may depend on gender, improvement in the severity of depressive symptoms, and protein concentrations. In the case of BDNF, its higher initial concentrations may provide better neuroregeneration (hence the positive correlation). On the other hand, resistin’s lower pre-treatment concentrations may imply a lower severity of inflammation, which is more easily overcome during treatment.

The results of most studies to date indicate that patients with depression are characterized by reduced adiponectin levels [23] regardless of the type of depression [46,47], although with lower baseline levels in women than men [48]. In this regard, the results we presented are consistent. We did not obtain statistical significance for the change in adiponectin concentrations after ECT treatment, probably because patients were taking pharmacotherapy before and during the study. It has been proven that antidepressants [49] and lithium [50] can lower adiponectin levels. Adiponectin has been identified as a predictor of antidepressant response to drugs of different mechanisms of action, such as ketamine, SSRIs, and SNRIs [49]. Our results also add non-pharmacological ECT treatment to this group.

### 5.2. Resistin

In the case of resistin, the main correlation observed was that the baseline concentration of this proinflammatory adipokine was significantly lower in patients with unipolar versus bipolar depression. We confirmed a significant correlation between proinflammatory resistin and anti-inflammatory adiponectin levels and that baseline resistin levels correlate with improvement in depressive symptoms. The observations are consistent with recent results from the Rahman et al., 2022 study [28]. The authors observed increased serum resistin levels in patients with major depressive disorder (MDD) compared to healthy controls and showed a significant correlation of serum resistin with HDRS score. The authors are inconclusive as to whether elevated resistin levels in depression are an effect or cause of the disease. Nonetheless, they suggest serum resistin as a potential candidate marker for depression because of its robust diagnostic performance and recommend further studies. Given the proinflammatory nature of resistin and its high baseline concentrations in depressed patients in the context of the inflammatory hypothesis, one would expect that therapy should result in a normalization (decrease) of its concentration. The nominal reduction of resistin concentration we observed, although it did not reach statistical significance, is consistent with predictions and previous observations. Weber-Hamann et al., 2007 observed a decrease in resistin in patients in remission after antidepressants; such a decrease was not observed in patients without remission [27]. The observation indicates that various mediating mechanisms, including HPA axis involvement, may influence resistin levels depending on the treatment used. The present study also replicates the results of our previous study of ECT in patients with bipolar depression [51]. At that time, we observed a decrease in resistin in bipolar depression types I and II starting with different baseline concentrations. In this study, we complement the results with observations from unipolar depression, which indicate different baseline resistin concentrations depending on the subtype of depression. It, therefore, appears that resistin may be a good marker for differential diagnosis.

### 5.3. BDNF

BDNF is a biomarker of enduring interest to researchers working on mental disorders. Its reduced concentrations are observed in most reports of depression, bipolar disorder, schizophrenia, and others [52,53]. It is indicated to be a good marker of disease status and course, as peripheral BDNF levels are reduced in exacerbations without significant differences in the euthymic state compared to control subjects. It is also a good predictor of treatment response. For example, antidepressant treatment increases serum BDNF levels in MDD responders and remissioners significantly more than in non-responders [54]. There is equally ample evidence of a link between BDNF and TRD. Meshkat et al. (2022) showed that of the three main therapies used for TRD (ECT, ketamine, and repetitive transcranial magnetic stimulation (rTMS)), it is ECT that has the most evidence of an increase in serum BDNF levels [55]. In our study, we observed a similar trend, which did not reach statistical significance. A possible explanation could be a ceiling effect, in which preceding drug therapy decreased baseline BDNF concentrations before ECT. Although the treatment improves the patient’s condition, we cannot demonstrate a significant relationship [56]. In conclusion, Meshkat et al. indicated that peripheral BDNF concentration increases after ECT but does not correlate with depressive symptoms and may not be a suitable predictor of treatment response in patients with TRD. Our results contradict these findings, as we demonstrated a significant association between pre-T BDNF concentrations and %HDRS score change. The higher the initial BDNF concentrations, the weaker the improvement in depressive symptoms. The fact that earlier studies failed to detect such a relationship may be due to the weak strength of this correlation and the difficulty in capturing it. 

Further studies of the proposed biomarkers make a lot of sense. In clinical practice, measurements of adipokines and BDNF before ECT could be used to predict clinical improvement and incorporate drugs leading to ECT potentiation or counteracting side effects. For example, if inflammation contributes to the pathogenesis of depression, anti-inflammatory drugs may be effective in treating depression. Theoretically, not only typical anti-inflammatory drugs can be used to treat depression but also cytokine receptor inhibitors, cytokine antibodies, and anti-inflammatory cytokines [57]. These drugs could also be used before ECT, similar to the previously tested ibuprofen (a non-steroidal anti-inflammatory drug) used to treat minor and moderate pain that prevented headaches after ECT [58].

## 6. Limitations

The present study’s authors are aware of the limitations, which include the lack of a healthy control group and relatively small subgroups of patients. However, the estimated minimum sample size (N = 48) and the determined effect sizes at the strong and very strong levels entitle us to a preliminary but reliable conclusion (Table S4). ECT initially induces a short-term inflammatory response in the body as part of an acute stress response. At the same time, repeated treatment generates a long-term down-regulation of the inflammatory and immune systems. Therefore, during a study, it may be essential to determine the concentration of adipokines and BDNF at several time points to capture the ultimate stabilization of their change. Also, regarding the extension of the biomarkers, the portfolio should be considered (e.g., the interleukins 6 (IL-6) and 1-beta (IL-1β), tumor necrosis factor-alpha (TNF-α), and C-reactive protein (CRP)) [59]. The strength of the presented study is the homogeneous group of patients with the control of health status, body weight, age, and medications. Although we have shown that the results obtained are generally consistent with previous findings, the limitations presented dictate that they be treated with some caution. In the case of TRD, by analogy with the pathophysiology of depression, it should be assumed that no single hypothesis explains all its symptoms. Most likely, we are dealing with multiple biological, genetic, and environmental mechanisms, prompting us to continue uncovering them [60].

## 7. Conclusions

Concentrations of adiponectin, resistin, and BDNF change after ECT treatment. These changes are correlated with improvements in the severity of depressive symptoms and are more or less expressed depending on the type of depression. For this reason, they remain exciting candidates as biomarkers in assessing the course of the disease and response to ECT treatment.

## Figures and Tables

**Table 1:** Inclusion and exclusion criteria.

Inclusion Criteria for the ECT Protocol		Exclusion Criteria for the Study
Adults of both sexes, aged ≥18		
Inpatients with diagnosed depression in the course of bipolar disorder in accordance with ICD-10 diagnostic criteria (diagnosis confirmed separately by two psychiatrists after a semi-structured interview)		Coexisting: schizophrenia or any psychotic features, depression in course of disorders other than bipolar disorders
No CNS diseases or injuries		Coexistence of serious general diseases such as epilepsy and other severe brain diseases (e.g., encephalitis, diseases with increased intracranial pressure, condition after a recent stroke) *
No cardiovascular disorders		Severe heart diseases (up to six months after myocardial infarction), significant hypertension, severe blood clotting disorders or significant anemia, aortic aneurysms, thrombophlebitis **
Good general somatic health		Presence of serious somatic disorders or unstable chronic illness, (e.g., advanced osteoporosis or some ophthalmic diseases), pregnancy or breastfeeding *
Qualification by anesthetist and cardiologist		Contraindications to ECT therapy *
Lack of overweight or obesity		BMI ≥ 25 **
Acceptance and informed consent to participate in the study		Lack of acceptance of study protocol and written informed consent
Current depressive episode, with 17-item HDRS score of at least 18		HDRS < 18
Treatment-resistant depression defined as lack of improvement after at least two courses of antidepressant and/or mood-stabilizing treatment (in optimal dosage and duration)		
Completion of treatment protocol		
Serum sample for laboratory tests		
Antidepressants discontinued during protocol	Lithium and mood-stabilizing antiepileptic drugs discontinued during protocol	

CNS: central nervous system, HDRS: Hamilton Depression Rating Scale, BMI: body mass index. * According to the recommendations of the Biological Section of the Polish Psychiatric Association. ** BMI of ≥25.0—overweight by the International Obesity Task Force [38].

**Table 2:** Demographic and clinical characteristics and biomarker concentrations in depression inpatients, before and after and electroconvulsive therapy.

		ECT (n = 54) Mean ± SD		
Age (years)		54.27 ± 12.80		
Onset of illness		39.96 ± 14.13		
Duration of illness (years)		14.49 ± 11.70		
Duration of current episode (weeks)		35.14 ± 65.69		
UD/BD		29/25		
Male/female		15/39		
Rec/non-rec		38/16		
	Pre-T Mean ± SD		Post-T Mean ± SD	*p* Value
		Mean ± SD		
HDRS	32.11 ± 6.19		12.09 ± 7.65	**<0.000** *
Adiponectin (ng/mL)	10,547.61 ± 7966.51		10,033.85 ± 6611.28	0.935
ΔAdiponectin (%)		7.31 ± 31.61%		
Resistin (ng/mL)	13.56 ± 3.44		12.45 ± 4.39	0.108
ΔResistin (%)		−7.09 ± 27.12%		
BDNF	12,064.34 ± 3535.29		12,438.60 ± 3594.63	0.942
ΔBDNF (%)		19.58 ± 93.02%		

* Pre-T: −95%CI = 30.42, +95%CI = 33.80 and post-T: −95%CI = 10.01, +95%CI = 14.18, r_c_ = 1.000; UD: unipolar depression; BD: bipolar depression; ECT: electroconvulsive therapy; pre-T: pre-treatment; post-T: post-treatment; rec: recovery; SD: standard deviation; results in bold mean statistically significant *p* value < 0.05; r_c_: Matched-pairs rank biserial correlation coefficient.

**Table 3:** Adipokines and BDNF concentration in unipolar vs. bipolar depression subgroups.

		BD (F31)	UD (F33)	*p* Value	r_g_
		Mean ± SD	Mean ± SD		
Pre-T	Adiponectin	9572.32 ± 6620.64	12,448.05 ± 10,413.88	0.800	
	Resistin	15.07 ± 3.20	11.67 ± 2.63	**0.023**	**0.620**
	BDNF	11,401.77 ± 3238.45	13,343.87 ± 3368.37	0.082	
Post-T	Adiponectin	9243.25 ± 5315.24	11,536.26 ± 8861.24	0.856	
	Resistin	14.06 ± 4.80	10.66 ± 1.92	**0.015**	**0.654**
	BDNF	12,198.65 ± 4369.07	12,738.59 ± 2566.85	0.660	
ΔAdiponectin		6.55 ± 27.05%	9.05 ± 41.67%	0.856	
ΔResistin		−4.90 ± 33.05%	−7.00 ± 14.31%	0.971	
ΔBDNF		26.05 ± 117.55%	1.92 ± 27.98%	0.853	

ECT: electroconvulsive therapy; pre-T: pre-treatment; post-T: post-treatment; SD: standard deviation; UD: unipolar disorder; BD: bipolar disorder; results in bold mean statistically significant *p* value < 0.05; r_g_: Glass rank biserial correlation coefficient.

**Table 4:** Adipokines and BDNF concentration in female vs. male subgroups.

		Female	Male	*p* Value	r_g_
		Mean ± SD	Mean ± SD		
Pre-T	Adiponectin	11,591.73 ± 7846.99	8310.21 ± 8359.85	0.091	
	Resistin	13.29 ± 3.13	14.15 ± 4.25	0.778	
	BDNF	12,250.67 ± 3957.42	11,579.87 ± 2111.30	0.364	
Post-T	Adiponectin	11,473.72 ± 7188.34	6948.43 ± 4015.38	**0.011**	**0.695**
	Resistin	12.78 ± 5.06	11.74 ± 2.64	0.481	
	BDNF	12,757.23 ± 3574.03	11,652.64 ± 3646.08	0.241	
ΔAdiponectin		6.37 ± 24.69%	9.32 ± 45.48%	1.000	
ΔResistin		−3.81 ± 29.91%	−14.11 ± 20.06%	0.205	
ΔBDNF		26.12 ± 107.69%	3.44 ± 36.20%	0.968	

ECT: electroconvulsive therapy; pre-T: pre-treatment; post-T: post-treatment; SD: standard deviation; results in bold mean statistically significant *p* value < 0.05; r_g_: Glass rank biserial correlation coefficient.

**Table 5:** Adipokines, BDNF concentration in recovered vs. non-recovered status subgroups.

		rec	Non-rec	*p* Value	r_g_
		Mean ± SD	Mean ± SD		
Pre-T	Adiponectin	11,576.54 ± 8112.95	4031.03 ± 498.75	**0.028**	**0.825**
	Resistin	13.31 ± 3.15	15.15 ± 5.55	0.774	
	BDNF	11,531.21 ± 3868.86	13,330.52 ± 2192.95	0.061	
Post-T	Adiponectin	10,695.16 ± 6899.17	5845.57 ± 300.94	**0.035**	**0.789**
	Resistin	13.03 ± 4.11	8.77 ± 5.20	0.151	
	BDNF	12,267.24 ± 4034.14	12,861.28 ± 2225.66	0.408	
ΔAdiponectin		1.21 ± 29.37%	45.96 ± 11.59%	**0.045**	**0.754**
ΔResistin		−1.55 ± 23.24%	−42.14 ± 26.96%	**0.028**	**0.825**
ΔBDNF		27.33 ± 109.10%	0.47 ± 19.08%	0.856	

ECT: electroconvulsive therapy; pre-T: pre-treatment; post-T: post-treatment; SD: standard deviation; results in bold mean statistically significant *p* value < 0.05; r_g_: Glass rank biserial correlation coefficient.

**Table 6:** Adipokines, BDNF concentration pre-T vs. post-T ECT in selected subgroups of patients.

	ECT Pre-T vs. Post-T		
		Z	*p* Value
Adiponectin			
	Total	0.08	0.935
	Male	0.17	0.866
	Female	0.23	0.820
	Rec	0.68	0.494
	Non-rec	1.60	0.109
	BD	0.07	0.944
	UD	0.07	0.944
Resistin			
	Total	1.61	0.108
	Male	1.86	0.063
	Female	0.74	0.460
	Rec	0.93	0.355
	Non-rec	1.34	0.181
	BD	0.70	0.485
	UD	1.33	0.183
BDNF			
	Total	0.07	0.942
	Male	0.11	0.910
	Female	0.04	0.970
	Rec	0.10	0.922
	Non-rec	0.34	0.733
	BD	0.44	0.657
	UD	0.03	0.974

ECT: electroconvulsive therapy; pre-T: pre-treatment; post-T: post-treatment; rec: recovery.

## Data Availability

The data that support the findings of this study are available upon reasonable request.

## Supplementary Materials

The following supporting information can be downloaded at: https://www.mdpi.com/article/10.3390/brainsci13101358/s1, Table S1. Correlation between adipokines, BDNF, and age of inpatients before and after electroconvulsive therapy (ECT); Table S2. Adipokines, BDNF concentration, and pharmacotherapy; Table S3. Association of adipokines, BDNF serum level with improvement in depressive symptoms; Table S4. The confidence intervals and effect size measures.
