# Supplementary material for: Changes in Adipokine, Resitin, and BDNF Concentrations in Treatment-Resistant Depression after Electroconvulsive Therapy
Source: Brain Sci. 2023 Sep 22;13(10):1358. doi: 10.3390/brainsci13101358 (PMC10605107; doi:10.3390/brainsci13101358)
Supplement: Supplementary file 1 [file brainsci-13-01358-s001.zip › brainsci-2556294-supplementary.pdf]

Table S1. Correlation between Adipokines, BDNF and age of inpatients before and after electroconvulsive therapy (ECT).

		Rs	*p*
Pre-T	Adiponectin	0.360	0.109
	Resistin	-0.160	0.489
	BDNF	0.079	0.580
Post-T	Adiponectin	0.374	0.095
	Resistin	-0.322	0.155
	BDNF	0.143	0.321
ΔAdiponectin		-0.247	0.281
ΔResistin		-0.142	0.540
ΔBDNF		0.094	0.517

ECT: electroconvulsive therapy; pre-T: pre-treatment; post-T: post-treatment; Rs: Spearman's rank correlation coefficient; p-value < 0.05

Table S2. Adipokines, BDNF concentration and pharmacotherapy.

		AN mean±SD	A mean±SD	AN+A mean±SD	p value
Pre-T	Adiponectin	8 492.45 ± 3 855.00	6 731.13 ± 3 910.11	11 858.95 ± 9 146.52	0.790
	Resistin	14.68 ± 2.76	13.95 ± 2.77	13.19 ± 3.81	0.591
	BDNF	10 564.77 ± 3 094.18	13 241.30 ± 1 944.08	12 514.29 ± 3 526.80	0.156
Post-T	Adiponectin	8 134.40 ± 3 516.01	5 805.27 ± 2 035.24	11 386.09 ± 7 449.90	0.300
	Resistin	14.47 ± 3.46	13.60 ± 0.79	11.68 ± 4.92	0.500
	BDNF	11 863.39 ± 3 704.32	13 816.52±2 838.86	12 380.29 ± 3 739.39	0.275
ΔAdiponectin		-1.16 ± 17.15%	0.03 ± 56.11%	11.03 ± 30.65%	0.737
ΔResistin		0.23 ± 2 4.42%	0.26 ± 22.45%	-10.50 ± 29.40%	0.634
ΔBDNF		41.70 ± 142.51%	8.80 ± 40.43%	9.12 ± 75.38%	0.974

ECT: electroconvulsive therapy; pre-T: pre-treatment; post-T: post-treatment; SD: standard deviation; AN: atypical neuroleptics; A: antidepressants

Table S3. Association of adipokines, BDNF serum level with improvement in depressive symptoms

	Adiponectin pre-T	Adiponectin post-T	ΔAdiponectin	Resistin pre-T	Resistin post-T	ΔResistin	BDNF pre-T	BDNF post-T	ΔBDNF
Rs	0.048	0.006	0.354	0.108	0.431	0.535	0.286	0.069	0.158
Rs^2	0.002	0.000	0.125	0.012	0.185	0.286	0.082	0.005	0.025
Rs^2 corr.	-0.048	-0.050	0.081	-0.038	0.145	0.250	0.064	-0.015	0.005
F(1,54)	0.045	0.001	2.858	0.238	4.555	8.014	4.619	0.241	1.277
p	0.834	0.979	0.106	0.631	**0.045**	**0.010**	**0.036**	0.625	0.264
S_e	0.227	0.227	0.212	0.226	0.205	0.192	0.211	0.218	0.216

pre-T: pre-treatment; post-T: post-treatment; results in bold means statistically significant p-value < 0.05; Rs: Spearman rank correlation coefficient

Table S4. The confidence intervals and effect size measures.

To Table 2

	pre-T		post-T			
	-95%CI	+95%CI	-95%CI	+95%CI	r_c	p
HDRS	30.42	33.80	10.01	14.18	1.000	**<0.001**
Adiponectin (ng/ml)	7015.5	14079.8	7102.6	12965.1	0.020	0.935
Resistin (ng/ml)	12.04	15.09	10.51	14.40	0.391	0.108
BDNF	11115.7	12966.6	11437.8	13439.4	0.012	0.942

To Table 3

		BD (F31)		UD (F33)			
		-95%CI	+95%CI	-95%CI	+95%CI	r_g	p
Pre-T	Adiponectin	5571.5	13573.1	3741.8	21154.3	0.077	0.800
	Resistin	13.14	17.00	9.47	13.88	0.62	**0.023**
	BDNF	10169.9	12633.6	11887.3	14800.5	0.285	0.082
Post-T	Adiponectin	6031.3	12455.2	4128.1	18944.4	0.058	0.856
	Resistin	11.16	16.96	9.06	12.27	0.654	**0.015**
	BDNF	10504.5	13892.8	11600.5	13876.7	0.075	0.660
ΔAdiponectin		-9.79	22.91	-25.81	43.89	0.058	0.856
ΔResistin		-24.86	15.06	-18.98	4.98	0.019	0.971
ΔBDNF		-19.53	71.63	-10.48	14.33	0.032	0.853

To Table 4

		Female		Male			
		-95%CI	+95%CI	-95%CI	+95%CI	r_g	p
Pre-T	Adiponectin	7246.2	15937.2	578.6	16041.8	0.467	0.091
	Resistin	11.56	15.02	10.22	18.08	0.09	0.778
	BDNF	10967.8	13533.5	10410.7	12749.1	0.162	0.364
Post-T	Adiponectin	7492.9	15454.5	3234.8	10662.0	0.695	**0.011**
	Resistin	9.98	15.59	9.30	14.18	0.200	0.481
	BDNF	11565.6	13948.9	9633.5	13671.8	0.211	0.241
ΔAdiponectin		-7.29	20.06	-32.74	51.37	0.010	1.000
ΔResistin		0.00	0.00	-32.69	4.46	0.352	0.205
ΔBDNF		-9.77	62.04	-16.61	23.49	0.007	0.968

To Table 5

		Rec		Non-rec			
		-95%CI	+95%CI	-95%CI	+95%CI	r_g	p
Pre-T	Adiponectin	7666.2	15486.9	2792.1	5270.0	0.825	**0.028**
	Resistin	11.79	14.83	1.37	28.94	0.123	0.774
	BDNF	10259.5	12802.9	12162.0	14499.1	0.327	0.061
Post-T	Adiponectin	7369.9	14020.5	5098.0	6593.2	0.789	**0.035**
	Resistin	11.05	15.02	-4.13	21.67	0.544	0.151
	BDNF	10922.2	13612.3	11628.8	14093.8	0.150	0.408
ΔAdiponectin		-12.94	15.37	17.20	74.73	0.754	**0.045**
ΔResistin		-12.75	9.65	-109.10	24.83	0.825	**0.028**
ΔBDNF		-9.04	63.71	-10.10	11.04	0.032	0.864

To Table 6

		pre-T				post-T					
		M	SD	-95%CI	+95%CI	M	SD	-95%CI	+95%CI	r_c	p
Adiponectin											
	Total	10547.6	7966.5	7015.5	14079.8	10033.9	6611.3	7102.6	12965.1	0.020	0.935

	Male	8310.2	8359.8	578.6	16041.8	6948.4	4015.4	3234.8	10662.0	0.071	0.866
	Female	11591.7	7847.0	7246.2	15937.2	11473.7	7188.3	7492.9	15454.5	0.067	0.820
	Rec	11576.5	8113.0	7666.2	15486.9	10695.2	6899.2	7369.9	14020.5	0.179	0.494
	Non-rec	4031.0	498.7	2792.1	5270.0	5845.6	300.9	5098.0	6593.2	0.772	0.109
	BD	9572.3	6620.6	5571.5	13573.1	9243.2	5315.2	6031.3	12455.2	0.033	0.944
	UD	12448.1	10413.9	3741.8	21154.3	11536.3	8861.2	4128.1	18944.4	0.056	0.944
Resistin											
	Total	13.57	3.44	12.04	15.09	12.45	4.39	10.51	14.40	0.391	0.108
	Male	14.15	4.25	10.22	18.08	11.74	2.64	9.30	14.18	0.786	0.063
	Female	13.29	3.13	11.56	15.02	12.79	5.06	9.98	15.59	0.217	0.460
	Rec	13.31	3.15	11.79	14.83	13.03	4.11	11.05	15.02	0.242	0.355
	Non-rec	15.16	5.55	1.37	28.94	8.77	5.19	-4.13	21.67	0.772	0.181
	BD	15.07	3.20	13.14	17.00	14.06	4.80	11.16	16.96	0.231	0.485
	UD	11.67	2.63	9.47	13.88	10.67	1.92	9.06	12.27	0.556	0.183
BDNF											
	Total	12041.1	3324.2	11115.7	12966.6	12438.6	3594.6	11437.8	13439.4	0.012	0.942
	Male	11579.9	2111.3	10410.7	12749.1	11652.6	3646.1	9633.5	13671.8	0.033	0.910
	Female	12228.2	3714.3	10989.7	13466.6	12757.2	3574.0	11565.6	13948.9	0.007	0.970
	Rec	11689.8	3795.0	10424.5	12955.1	12267.2	4034.1	10922.2	13612.3	0.018	0.922
	Non-rec	12907.8	1445.6	12107.3	13708.4	12861.3	2225.7	11628.8	14093.8	0.100	0.733
	BD	11606.7	3100.5	10404.5	12808.9	12198.7	4369.1	10504.5	13892.8	0.099	0.657
	UD	13056.3	3145.4	11661.7	14450.9	12738.6	2566.9	11600.5	13876.7	0.012	0.974
